# High Ligation of the Inferior Mesenteric Artery Induces Hypoperfusion of the Sigmoid Colon Stump During Anterior Resection

**DOI:** 10.3389/fsurg.2021.756873

**Published:** 2021-12-13

**Authors:** Jun Higashijima, Toru Kono, Mitsuo Shimada, Ayumu Sugitani, Hideya Kashihara, Chie Takasu, Masaaki Nishi, Takuya Tokunaga, Kozo Yoshikawa

**Affiliations:** ^1^Department of Digestive Surgery and Transplantation, Institute of Health Biosciences, The University of Tokushima, Tokushima, Japan; ^2^Center for Clinical and Biomedical Research, Sapporo Higashi Tokushukai Hospital, Sapporo, Japan; ^3^Advanced Surgery Center, Sapporo Higashi Tokushukai Hospital, Sapporo, Japan

**Keywords:** anterior rectal resection, ileocecal resection, hypoperfusion, artery ligation, anastomotic leakage

## Abstract

**Background:** Anastomotic leakage (AL) after colorectal surgery is associated with insufficient vascular perfusion of the anastomotic ends. This study aimed to evaluate the effect of high vs. low ligation of the ileocolic artery and inferior mesenteric artery, respectively, on the vascular perfusion of the bowel stumps during ileocecal resection (ICR) and anterior rectal resection (AR).

**Methods:** We retrospectively evaluated patients who underwent ICR or AR between 2016 and 2020. Real-time indocyanine green fluorescence angiography was performed to measure the fluorescence time (FT) as a marker of the blood flow in the proximal and distal stumps before anastomosis.

**Results:** Thirty-four patients with lower right-sided colon cancer underwent laparoscopic ICR. Forty-one patients with rectosigmoid colon or rectal cancer underwent robotic high AR (HAR) (*n* = 8), robotic low AR (LAR) (*n* = 6), laparoscopic HAR (*n* = 8), or laparoscopic LAR (*n* = 19). The FT was similar in the ileal and ascending colon stumps (*p* = 1.000) and did not differ significantly between high vs. low ligation of the ileocolic artery (*p* = 0.934). The FT was similar in the sigmoid colon and rectal stumps (*p* = 0.642), but high inferior mesenteric artery ligation significantly prolonged FT in the sigmoid colon during AR compared with low ligation (*p* = 0.004), indicating that the high ligation approach caused significant hypoperfusion compared with low ligation. The AL rate was similar after low vs. high ligation.

**Conclusions:** Low vascular perfusion of the bowel stumps may not be an absolute risk factor for AL. High inferior mesenteric artery ligation could induce sigmoid colon stump hypoperfusion during anterior rectal resection.

## Introduction

Anastomotic leakage (AL) is a serious complication of colorectal surgery. The AL rate in colorectal surgery is typically higher after rectal resection than after right-sided colon resection, although AL is affected by numerous risk factors (1–6). Insufficient blood supply at the proximal and/or distal anastomotic ends inevitably contributes to AL; however, the etiology of AL has not been fully clarified. There is no information about the differences in the vascular perfusion of the bowel stumps for anastomosis after ligation of the ileocolic artery (ICA) vs. ligation of the inferior mesenteric artery (IMA).

Intraoperative judgment by the operating surgeon may lead to an underestimation of the risk of AL based on visual inspection (7, 8). Therefore, real-time indocyanine green (ICG) fluorescence angiography (ICGFA) was developed to evaluate intestinal perfusion. ICGFA is useful for confirming vascular perfusion of the intestines at the site of anastomosis (9–12).

The aim of the present study was to analyze our experience using intraoperative ICGFA to evaluate the vascular perfusion of the bowel stumps (proximal and distal) for anastomosis during ileocecal resection (ICR) with high or low ligation of the ICA and during anterior rectal resection (AR) with high or low ligation of the IMA.

## Methods

**Figure 1:**
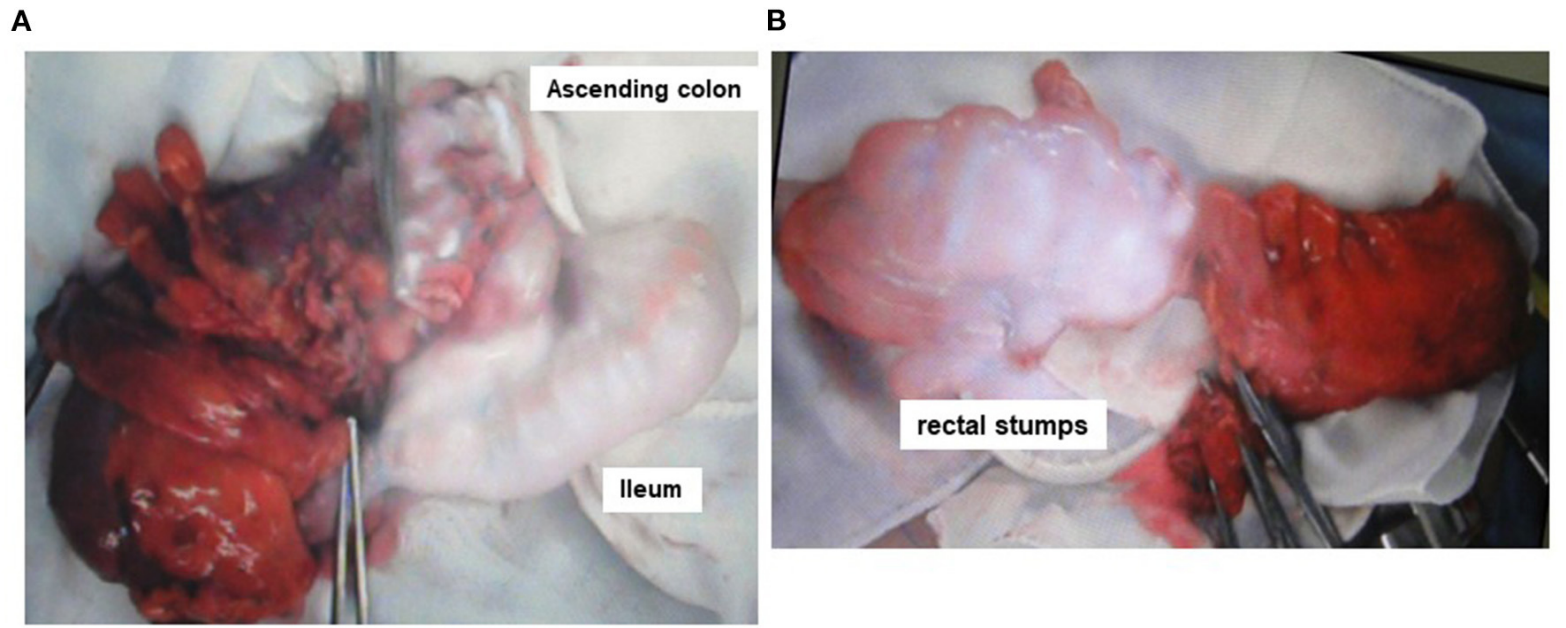
Intraoperative images of **(A)** ileocecal resection and **(B)** anterior rectal resection.

In this retrospective study, we evaluated the medical records of patients who underwent ICR or AR at Tokushima University Hospital between January 2016 and December 2020. During this period, 34 consecutive patients with cecal or ascending colon cancer underwent ICR, and 41 consecutive patients with rectosigmoid colon or rectal cancer underwent AR without diverting ileostomy. Representative intraoperative images of ICR and AR are shown in Figures 1A,B, respectively. The exclusion criteria were a history of major gastrointestinal surgery, a history of adverse reaction to ICG and/or iodine, pregnancy and/or lactation, age ≥ 80 years, neoadjuvant chemotherapy, radiation therapy, and clinical stage IV cancer. The diagnoses of cancer were made based on conventional clinical, radiological, endoscopic, and histopathological criteria. High ligation of the IMA (H-IMA) was defined as ligation above the left colic artery, while high ligation of the ICA (H-ICA) was defined as the situation in which the ICA traveled anterior and superior to the superior mesenteric vein in ICR. The definition of AL included the passage of fecal material from the drain, formation of a pelvic abscess, or peritonitis. The diagnosis of AL was usually confirmed by clinical findings and abdominal computed tomography.

The study was approved by the institutional review board of Tokushima University Hospital (approval no. ToCMS 3215-2) and was performed in accordance with the ethical standards laid down in the Declaration of Helsinki. All included participants provided written informed consent.

The blood flow in the oral and anal stumps was evaluated using ICGFA (Diagnogreen; Daiichi Sankyo Co., Ltd., Tokyo, Japan) prior to completion of the anastomosis. Intravenously injected ICG emits light with a peak wavelength of 800–850 nm. The ICG was visualized in the blood vessels after excitation with near-infrared light (760–780 nm) using the Hyper Eye Medical System (Mizuho Medical Co., Ltd. Tokyo, Japan). The fluorescence time (FT) in each stump was measured using the Hyper Eye Medical System. The FT was defined as the time from ICG injection and flushing of the injection route to the timepoint at which the strongest fluorescent signal was observed in the stump (12).

GraphPad Prism software version 6.0 for Windows (San Diego, California, USA) and SAS Studio Release 3.6 (SAS Institute Inc., Cary, NC, USA) were used to create the graphs and perform the calculations and statistical analyses. Continuous variables were compared among the multiple groups using one-way analysis of variance testing. When a variable significantly differed between groups, Tukey's multiple comparisons method was adopted for intergroup analysis. Categorial outcomes were compared among the multiple groups using Fisher's exact test. Data are presented as the mean ± standard deviation. All tests were two-sided, and the level of statistical significance was set at *p* < 0.05.

## Results

The demographic details and characteristics of patients with lower right-sided colon cancer, rectosigmoid colon cancer, or rectal cancer are presented in Table 1. There were no intraoperative adverse events or conversions to open surgery. All patients with lower right-sided colon cancer (*n* = 34) underwent laparoscopic ICR. Fourteen patients with rectosigmoid colon cancer underwent robotic high AR (HAR) (*n* = 8) or low anterior resection (LAR) (*n* = 6), while 27 patients with rectal cancer underwent laparoscopic HAR (*n* = 8) or LAR (*n* = 19). There were no adverse reactions related to the injection of ICG. ICG-enhanced fluorescence was detected in 100% of the patients. No changes in surgical plan occurred for any patient before or after ICGFA.

**Table 1:** Demographic details and clinical characteristics of patients with lower right-sided colon cancer and patients with rectosigmoid colon or rectal cancer.

		**Lower right-sided colon cancer (*n =* 34)**	**Rectosigmoid colon or rectal cancer (*n =* 41)**	***P*-value**
Gender, male	*n* (%)	15 (44.1)	21 (51.2)	*P =* 0.6440
Age, years	median (IQR)	69 (61−73)	67 (59-71)	*P* = 0.1171
**Location**				
Cecum	*n* (%)	12 (35.3)		
Ascending colon	*n* (%)	22 (64.7)		
Rectosigmoid colon	*n* (%)		24 (58.5)	
Rectum	*n* (%)		17 (41.5)	
**Pathological stage**								*P* = 0.6492
0	*n* (%)	3 (8.8)	1 (2.4)	
I	*n* (%)	14 (41.2)	17 (41.5)	
II	*n* (%)	7 (20.6)	11 (26.8)	
III	*n* (%)	10 (29.4)	12 (29.3)	
**Lymph node dissection**								*P* = 0.0496
D2	*n* (%)	15 (44.1)	9 (22.0)	
D3	*n* (%)	19 (55.9)	32 (78.0)	
High ligation^*^				
Ileocolic artery	*n* (%)	19 (55.9)		
Inferior mesenteric artery	*n* (%)		31 (75.6)	
**Type of approach**								*P* <0.0001
Laparoscopic	*n* (%)	34 (100)	27 (65.9)	
Robotic	*n* (%)	0 (0)	14 (34.1)	
**Type of resection**				
Ileocaecal resection	*n* (%)	34 (100)		
High anterior resection	*n* (%)		16 (39.0)	
Low anterior resection Type of anastomosis	*n* (%)		25 (61.0)	
**Type of anastomosis**								*P* = 0.0067
Stapled anastomosis	*n* (%)	28 (82.4)	41 (100)	
Hand sawn anastomosis	*n* (%)	6 (17.6)	0 (0)	
Anastomotic leakage	*n* (%)	0 (0)	3 (7.7)	
Follow up 30 d mortality	*n* (%)	0 (0)	0 (0)	

* *High ligation: ligation of the inferior mesenteric artery proximal to the left colic artery in anterior resection/ligation of the ileocolic artery as the situation in which the ileocolic artery travels anterior and superior to the superior mesenteric vein in ileocecal resection*.

*Each P-value was calculated using Fisher's exact test or t-test (age). IQR, interquartile range*.

### Ileocecal Resection

**Figure 2:**
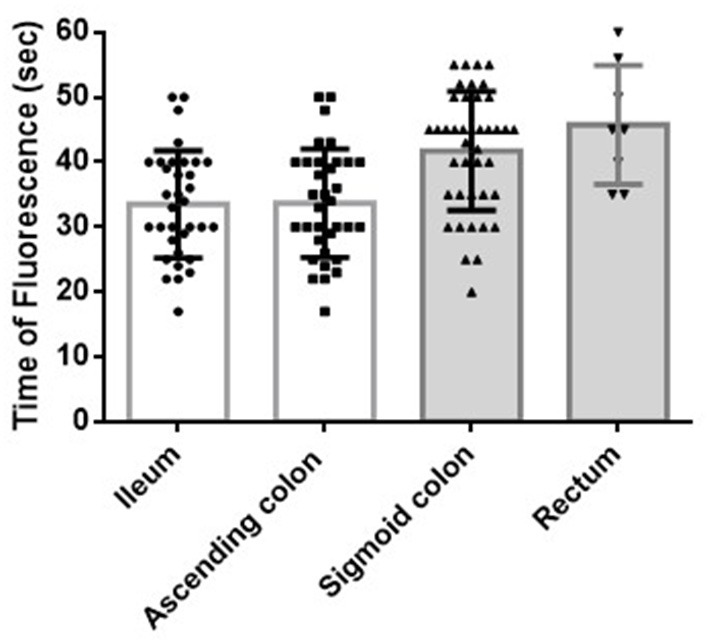
Blood flow of the bowel stumps during ICR or AR. The FT of the ileal and ascending colon stumps during ICR was 33.5 ± 8.1 s and 33.7 ± 8.2 s, respectively (*p* = 1.000). The FT of the sigmoid colon and rectal stumps during AR was 41.8 ± 9.1 s and 45.8 ± 9.3 s, respectively (*p* = 0.642). The FTs during AR (of both the sigmoid colon and rectal stumps) were significantly longer than the FTs during ICR (of both the ascending colon and ileal stumps: sigmoid colon vs. ascending colon, *p* = 0.001; sigmoid colon vs. ileum, *p* = 0.001; rectum vs. ascending colon, *p* = 0.003; rectum vs. ileum, *p* = 0.003). ICR: ileocecal resection; AR: anterior rectal resection; FT: fluorescence time (time from indocyanine green injection and injection route flush to the time at which the strongest fluorescent signal was observed in the stump).

**Figure 3:**
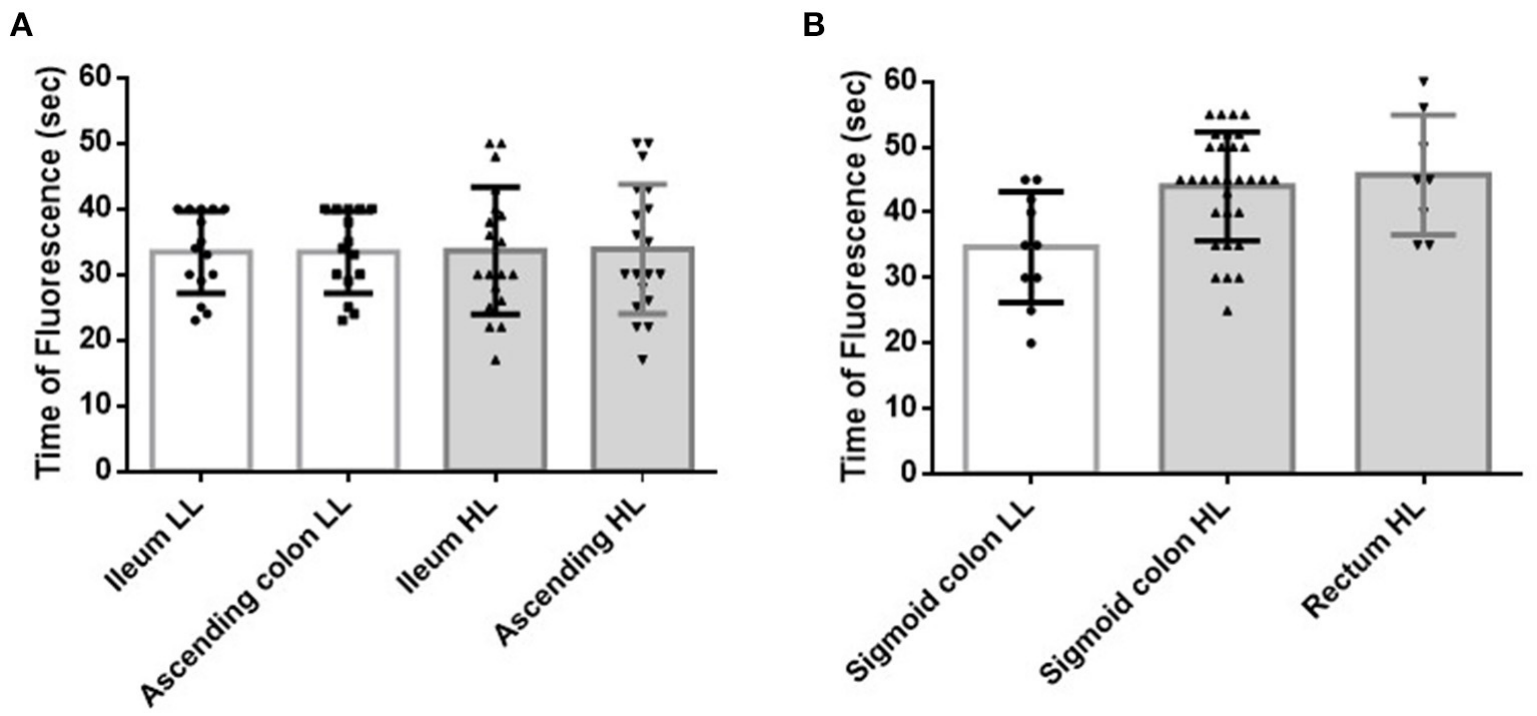
Effect of the level of ligation of the main artery on the blood flow of the bowel stumps during surgery. **(A)** Effect of the level of ligation of the ICA during ICR. HL of the ICA did not affect the FT of the ileal or ascending colon stumps (*p* = 0.860 and *p* = 0.934, respectively). **(B)** Effect of the level of ligation of the IMA during AR. HL of the IMA significantly prolonged the FT of the sigmoid colon (*p* = 0.004). All patients with a measurable rectal stump blood flow received HL of the IMA. ICA, ileocecal artery; ICR, ileocecal resection; HL, high ligation; FT, fluorescence time (time from indocyanine green injection and injection route flush to the time at which the strongest fluorescent signal was observed in the stump); IMA, inferior mesenteric artery; AR, anterior rectal resection; LL, low ligation.

The FTs of the ileal and ascending colon stumps during ICR were 33.5 ± 8.1 s and 33.7 ± 8.2 s, respectively (*p* = 1.000) (Figure 2). H-ICA did not affect the FT of the ileal and ascending colon stumps compared with low ligation of the ICA (L-ICA; *p* = 0.860 and *p* = 0.934, respectively) (Figure 3A). No AL was observed after ICR.

### Anterior Rectal Resection

The FTs of the sigmoid colon and rectal stumps during AR were 41.8 ± 9.1 s and 45.8 ± 9.3 s, respectively (*p* = 0.642) (Figure 2). The FT of the sigmoid colon was significantly longer after H-IMA compared with after low ligation of the IMA (L-IMA; *p* = 0.004) (Figure 3B). All patients with a measurable rectal stump blood flow underwent H-IMA.

**Figure 4:**
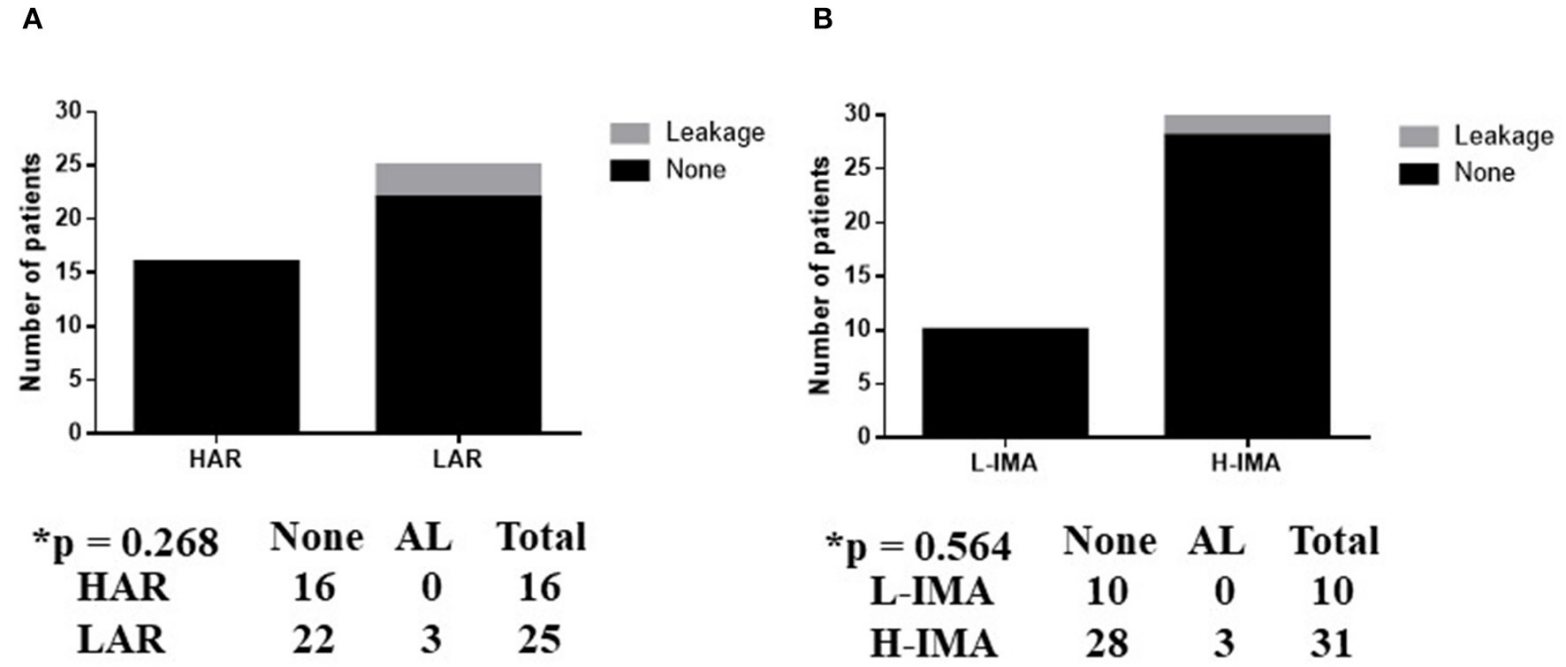
**(A,B)** Association between anastomotic leakage in anterior rectal resection and surgery type. L-IMA, low ligation of the IMA; H-IMA, high ligation of the IMA; LAL, low anterior rectal resection; HAR, high anterior rectal resection. Calculated by Fisher's exact 2 × 2 contingency table.

AL was observed in three patients (one male and two females) after LAR, while no AL occurred after HAR in any patients; this difference was not statistically significant (*p* = 0.268) (Figure 4A). The level of ligation of the IMA was not associated with the rate of AL (*p* = 0.564) (Figure 4B). All patients with AL underwent re-operation for drainage.

### Ileocecal Resection vs. Anterior Rectal Resection

The FT during AR (of both the sigmoid colon and rectal stumps) was significantly longer than the FT during ICR (of both the ascending colon and ileal stumps: sigmoid colon vs. ascending colon, *p* < 0.001; sigmoid colon vs. ileum, *p* < 0.001; rectum vs. ascending colon, *p* = 0.003; rectum vs. ileum, *p* = 0.003) (Figure 2).

**Figure 5:**
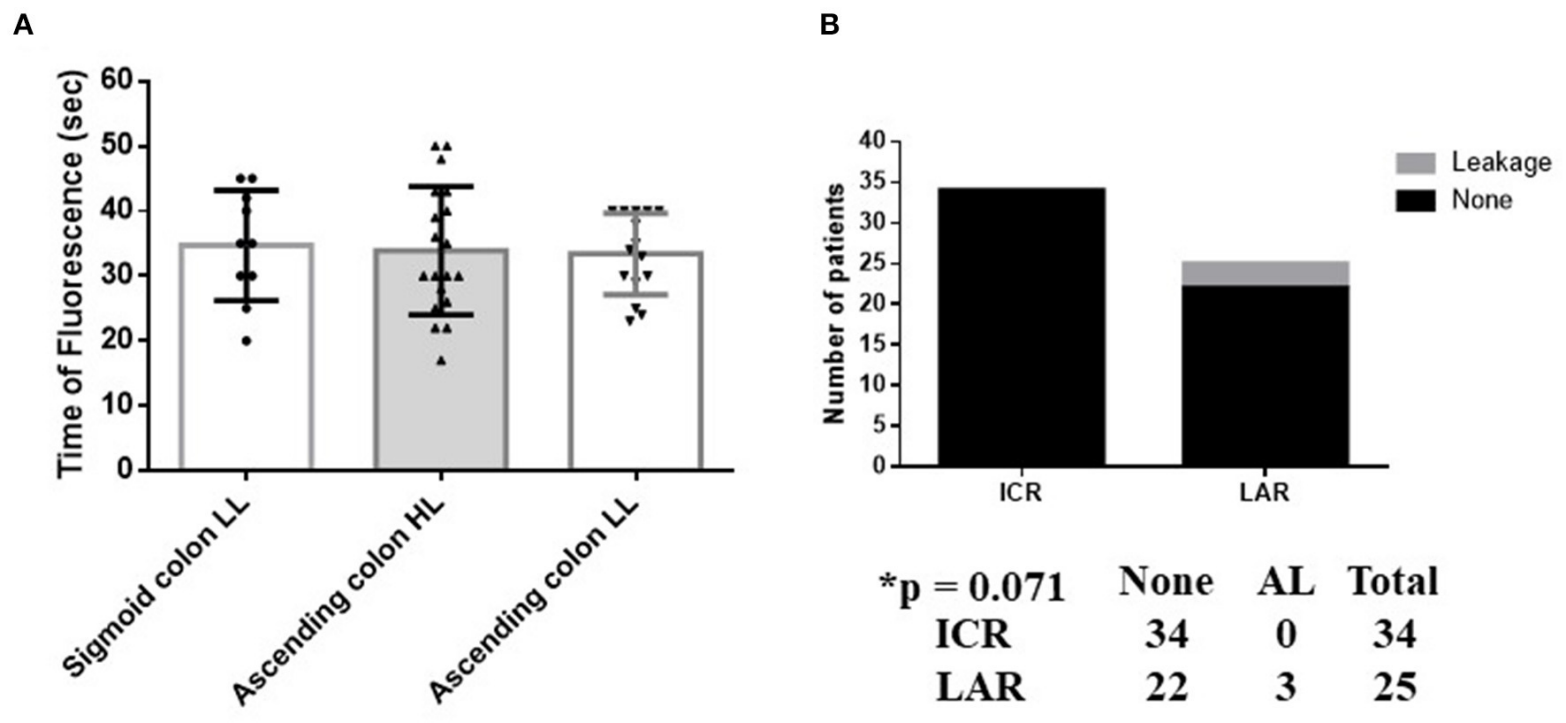
Blood flow and anastomotic leakage in ICR vs AR. **(A)** Comparison of the FT between the ascending and sigmoid colon with preservation of the left colic artery. The FT of the sigmoid colon with LL of the IMA (34.7 ± 8.5 s) is similar to the FT of the ascending colon with HL or LL of the ICA (33.9 ± 9.9 s and 33.4 ± 6.3 s, respectively) (*p* = 0.932). **(B)** Association between AL and surgery type. The occurrence of AL in LAL did not represent a significant difference vs. ICR (*p* = 0.071). Calculated by Fisher's exact 2 × 2 contingency table. HL, high ligation; LL, low ligation; ICR, ileocecal resection; LAL, low anterior rectal resection.

The FT of the sigmoid colon after L-IMA (34.7 ± 8.5 s) was similar to the FT of the ascending colon after H-ICA or L-ICA (33.9 ± 9.9 s and 33.4 ± 6.3 s, respectively) (*p* = 0.932) (Figure 5A).

Patients who underwent LAR did not exhibit a significantly different prevalence of AL to those who underwent ICR (*p* = 0.071) (Figure 5B).

## Discussion

We limited the surgical technique in our investigation to ICR and AR to unify measurements in a specific region of the intestinal tract. This limitation minimized variability between the two study groups. In our study, intraoperative ICGFA revealed that H-IMA (above the left colic artery) caused significant hypoperfusion at the sigmoid colon stump during AR compared with L-IMA. This might partially explain the results of a previous prospective study of 616 patients, in which patients with H-IMA had a 3.8-fold higher prevalence of AL after AR than those without H-IMA (13). Compared with H-IMA, L-IMA is reportedly associated with a lower risk of AL during curative resection of the sigmoid colon in rectal cancer (14) and laparoscopic radical resection of rectal cancer (15). In contrast, a randomized study revealed that the IMA ligation level was unrelated to AL after AR (16). Moreover, a recent systematic and meta-analysis study demonstrated that the rate of AL did not significantly differ between the L-IMA and H-IMA groups (8.6% vs. 13.2%, respectively) (17), which is compatible with our results, in which the AL rate was similar after low ligation (0%) vs. high ligation (12.5%, *p* = 0.564). Overall, the association between the level of ligation of the IMA and the incidence of AL after AR remains controversial. However, these previous studies did not intraoperatively measure the blood flow of the bowel stumps involved in anastomosis. The present preliminary study findings suggest that the blood flow of the bowel stumps requiring anastomosis is not an absolute risk factor for AL but does contribute to anastomotic healing. Unfortunately, we did not investigate the effect of the level of ligation of the IMA on the FT of the distal rectal stump. Because the perfusion of both the proximal and distal stumps affects the success of anastomosis, the FT of the distal stump warrants investigation in a future study.

Intraoperative judgment by the operating surgeon may be subjective and lead to an underestimation of the risk of AL based on visual inspection (7, 8). Therefore, various techniques have been developed to evaluate intestinal perfusion, such as ICGFA and laser Doppler flowmetry (18–20). Several studies using laser Doppler flowmetry have shown that sigmoid colon blood flow is significantly lower after H-IMA compared with L-IMA during sigmoid colon and rectal resection (21–23). ICGFA, intraoperative Doppler ultrasound, laser Doppler flowmetry, and oxygen spectroscopy have not been widely used to evaluate intestinal perfusion because these techniques cannot be easily applied during surgery. However, ICGFA has shown potential as a tool with which to assess bowel perfusion and it may contribute to reducing the risk of AL in colorectal surgery (9, 11, 17), although this has mainly been shown in cohort studies with insufficient levels of evidence. A randomized controlled trial reported that the bowel resection was extended because of insufficient perfusion of the colon stump in 11% of patients undergoing colorectal surgery (24); however, the AL rate was similar in the ICGFA group (5%) and the control group (9%). Thus, the contribution of ICGFA to the reduction of AL in colorectal surgery via an intraoperative change in the surgical plan remains controversial.

The effect of ICGFA on the risk of colorectal AL has only been assessed based on the perfusion of the proximal bowel in most studies. However, the achievement of successful anastomotic healing requires good perfusion of the bowel stumps on both sides. Therefore, ICGFA must be used to assess both the distal and proximal bowel stumps to evaluate the correlation between the bowel perfusion and the rate of AL. The present preliminary study assessed both the proximal and distal bowel stumps after bowel resection. However, it is difficult to estimate the blood perfusion of the distal rectal stump because the pelvic space is too small to appropriately position the instruments without disturbing the surrounded organs, especially if fluorescence of the small intestine occurs at the same time. In the present study, the blood flow of the rectal stump was measured in only eight subjects (19.5%). Larger numbers of patients should be included in future studies using ICGFA to assess the distal stump perfusion.

With H-IMA, the remnant proximal colon is supplied by the middle colic artery and relies only on marginal blood flow, and eventually Riolan arcades (<10%) (25–27). In a recent study, digital subtraction angiography revealed that one-third of 154 patients >65 years with various diseases had no blood supply at or below the splenic flexure because of the absence of the left colic artery, hypoperfusion of the marginal artery, or history of atherosclerosis (28). This might explain why the level of ligation of the IMA affected the perfusion of the sigmoid colon during AR in the present study. However, we did not evaluate the perfusion region of the whole colorectum before surgery. Both the preoperative identification of the IMA branching pattern and perfusion region and the intraoperative information regarding the perfusion of the bowel stumps prior to anastomosis might be used to implement changes in surgical plans for patients at high risk of AL.

The AL rate varies widely and depends on the anatomic location of the anastomosis. The reported AL rate after laparoscopic ICR and right hemicolectomy ranges from 1 to 2.6% (29–31), whereas the AL rate after only laparoscopic AR without defunctioning stoma ranges from 6% to more than 10% (32–34). In the present study, the AL rate was 7.7% after AR, which is similar to the rate reported in previous studies. The AL rate seemed to be highest after rectal resection, particularly after LAR, although the precise reason is not clear. In the present study, all patients with AL had undergone LAR. The distal rectum is supplied by both the middle rectal artery and the lower rectal artery after HAR, whereas the distal rectum is only supplied by the lower rectal artery after LAR, making it more difficult to maintain an adequate blood supply (25–27). Additionally, the dorsocaudal rectal ampulla frequently contains a vessel-deficient area that cannot be compensated for by another vessel that supplies the rectum (35). This might explain why the AL rate was higher after LAR than HAR.

There are several limitations to our study. First, it was a retrospective study. Second, a very small sample size was examined, and thus our observations should be confirmed in a large prospective study population.

In conclusion, intraoperative ICGFA revealed that H-IMA could induce hypoperfusion of the sigmoid colon stump during AR. Future studies are required to confirm the effects of high vs. low ligation of the ileocolic artery and inferior mesenteric artery, respectively, on AL following ICR and AR.

## Data Availability Statement

The raw data supporting the conclusions of this article will be made available by the authors, without undue reservation.

## Ethics Statement

The studies involving human participants were reviewed and approved by the institutional review board of Tokushima University Hospital (approval No. ToCMS 3215-2). The patients/participants provided their written informed consent to participate in this study.

## Author Contributions

All authors listed have made a substantial, direct, and intellectual contribution to the work and approved it for publication.

## Conflict of Interest

The authors declare that the research was conducted in the absence of any commercial or financial relationships that could be construed as a potential conflict of interest.

## Publisher's Note

All claims expressed in this article are solely those of the authors and do not necessarily represent those of their affiliated organizations, or those of the publisher, the editors and the reviewers. Any product that may be evaluated in this article, or claim that may be made by its manufacturer, is not guaranteed or endorsed by the publisher.
